# Application of Information Technologies and Programming Methods of Embedded Systems for Complex Intellectual Analysis

**DOI:** 10.3390/e23010094

**Published:** 2021-01-11

**Authors:** Vitalii Emelianov, Nataliia Emelianova, Anton Zhilenkov, Sergei Chernyi

**Affiliations:** 1 Department Business Informatics, Financial University under the Government of the Russian Federation, 49 Leningradsky Prospekt, 125993 Moscow, Russia; inter@fa.ru (V.E.); n.yemelianova@gmail.com (N.E.); 2 Department of Cyber-Physical Systems, St.Petersburg State Marine Technical University, 190121 St. Petersburg, Russia; zhilenkovanton@gmail.com; 3 Department Complex Information Security, Admiral Makarov State University of Maritime and Inland Shipping, 198035 Saint-Petersburg, Russia; 4 Department of Ship’s Electrical Equipment and Automatization, Kerch State Maritime Technological University, 298309 Kerch, Russia

**Keywords:** intelligent system, metallographic analysis, software, neural networks, expert subsystem

## Abstract

An information model is outlined, which represents an intelligent system of metallographic analysis in the form of a set of subsystems, the interaction of which ensures the performance of metallographic analysis functions. The structure of the information storage subsystem for metallographic analysis is presented. The deployment model of an intelligent metallographic analysis system is proposed and described. The paper outlines the approach to the presentation of an expert subsystem for metallographic quality control of metals based on a neural network. The process of finding a close precedent in metallographic analysis with reference to a multilayer neural network is described. An intelligent metallographic analysis system is described, which based on proposed information model. A specialized software of an intelligent metallographic analysis system is presented. The functioning results of the developed system for processing images of steel microstructures to determine the steel quantitative parameters is presented.

## 1. Introduction

Modern metallurgical production is characterized by an increase in the requirements for failure-free functioning of critical production facilities and a reduction in the cost of repairs and accident handling [1].

It is impossible to improve the quality of national machine engineering products and to reduce their costs without improving existing methods of metal quality control. The use of modern approaches based on the use of information technology makes it feasible to increase the accuracy and efficiency of product quality control [2,3]. One of the main methods for controlling the quality of metal in production is metallographic analysis [4,5,6]. To date, the level of automation of the central factory laboratories of metallographic quality control of metals is insufficient [5,7,8,9]. It suggests that at the moment the task of creating an automated system of metallographic quality control of metals should be urgent.

To create a system of this kind, it is necessary to develop an information model that would display its main subsystems and components, their information flows and the order of their interaction.

Currently, it is difficult to imagine any kind of production that lacks automation elements. Each plant, factory strives to increase its efficiency, improve the quality of products and minimize costs. In addition, automation systems are able to protect expensive equipment by turning it off in the event of an accident.

## 2. Information Model of the Intelligent Metallographic Analysis System

The information model can be described from a morphological, functional and information points of view [10,11]. According to the decomposition principle [12], the developed system can be represented as a set of subsystems, the interaction of which will ensure the performance of the required operations.

Let us consider the subsystems as generalized information converters that implement a certain set of functions for compiling an informational description of metal microstructure images:

(1) $$Functions=\left\{f_{1},f_{2},f_{3},f_{4},f_{5}\right\},$$

where $f_{1}$ is the function responsible for automatic registration of metal images; $f_{2}$ is the automatic processing of a metal image; $f_{3}$ is the function responsible for metal data analysis; $f_{4}$ is the function responsible for the database creation and a knowledge database by registering all the parameters of every image of the metal microstructure and $f_{5}$ is the function responsible for communicating with a central database of an automated system to support distributed databases.

Based on the principles of integrating problematic, attributive and situational information components proposed in [13], it seems possible to determine the complete flow of the system information as follows:

Since the implementation of the function $f_{1}$ to one degree or another solves the problem of automatic control of the quality of metals, the component of the information generated by the function $f_{1}$ (microstructure image of the metal) is defined as the problematic component of the complete information and is designated as *I_P_*.

Functions $f_{2}$ and $f_{3}$ make up the attributive component of the information description which is expressed as *I_A_*.

Information generated by functions $f_{4}$ and $f_{5}$ is not associated with the characteristics of the metal microstructure images. It is defined as a situational component and expressed as *I_S_*.

Thus, the complete information the system generates is expressed as the sum of the three components:

(2) $$I=I_{P}+I_{A}+I_{S}.$$

**Figure 1:**
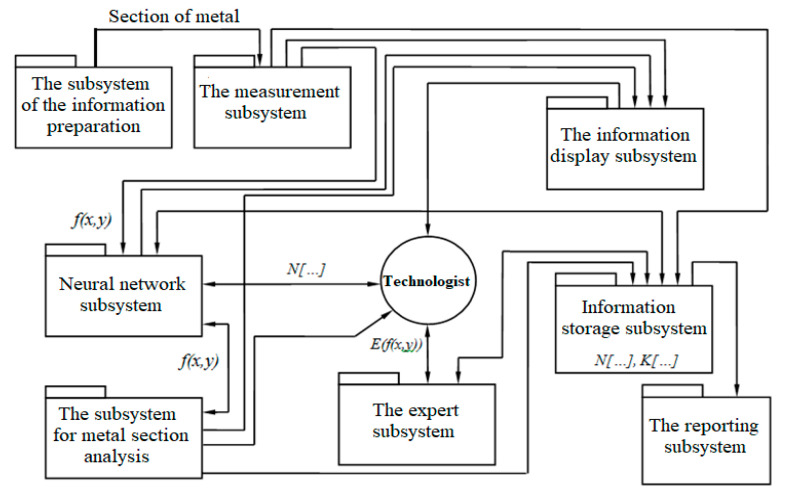
Decomposition of the intelligent metallographic analysis system.

The implication is that the system can be divided into subsystems not only in terms of functionality, but also in the types of information generated, which will optimize the distribution of information flows. Thus, the architecture of an intelligent metallographic analysis system includes the following subsystems (Figure 1).

The information preparation subsystem is a specialized tool for preparing a metal sample for metallographic analysis.

The measurement subsystem is a means of imaging the microstructure of metals. This subsystem implements the function $f_{1}$.

Information display subsystem is the means for visualizing the process of metallographic analysis and its outcomes.

The neural network subsystem, together with the expert subsystem, implements the function $f_{3}$. The subsystem is responsible for the implementation of neural network models for determining the quantitative characteristics of metals.

The microstructure analysis subsystem is a subsystem that implements the function $f_{2}$, intended for preliminary processing of images of metal microstructures.

The information storage subsystem is a repository of input, intermediate and output data generated in the process of metallographic analysis. The functions $f_{4}$ and $f_{5}$ are assigned to this subsystem.

The reporting subsystem is a module for creating reports of various types based on the results of metallographic analysis.

The intelligent system, built on the proposed model, performs as follows:

(1) After preparation, the metal sample under analysis is placed on the desktop of the measurement subsystem (microscope). The digital video camera receives the image *P*(*x*, *y*) and transfers it to the computer in the digitized form as a stream of video information.
(2) This stream goes to the input of specialized software. Since certain requirements are imposed on the image, which it should comply with, before further actions, the processing module makes changes to the image structure:

(3) $$f\left(x,y\right)=F_{PR}\left(P\left(x,y\right)\right)$$

Afterwards the calculation of the information features of the image is performed:

(4) $$P_{x,y}=\left\{\sin\left(A\right),\cos\left(A\right),G_{p}\right\}$$

where $P_{x,y}$ is a set of parameters characterizing the asserted base point of the image. This set is calculated by the Prewitt’s filter formulas [14].

(3) Next, the image characteristics are sent to the input *X_NN_* of the neural network subsystem. The neural network module analyzes them and generates a recognition result *Y_NN_*:

(5) $$X_{NN}=\overset{n}{\underset{i=1}{\cup }}\left\{\sin\left(A_{i}\right),\cos\left(A_{i}\right),G_{p_{i}}\right\},$$

(6) $$Y_{NN}=f\left(X_{NN}\right).$$

The result, together with the recognized image, is sent to the data processing and storage server (data storage subsystem). The operator and (or) the technologist has the opportunity in real time to monitor the process.

(4) In addition, the server accumulates the results of performance and by means of an expert subsystem allows one to evaluate the properties of the metal:

(7) $$\left(\sigma_{B_{NOMINAL}},\sigma_{T_{NOMINAL}},\sigma_{5_{NOMINAL}}\right)\to \left(\sigma_{B},\sigma_{T},\sigma_{5}\right),$$

where $\sigma_{B}$ is the metal ultimate tensile strength; $\sigma_{T}$ is the metal flow stress; and $\sigma_{5}$ is the metal percentage elongation.

Based on the assessed properties, the system generates recommendations and provides decision support regarding the metal purpose group. Also, the technologist, using the section processing subsystem, has the capability to process the image of the microstructure on his or her own. Neural network experience is stored in a database. The resulting solution is also sent to the database for further storage.

(5) After processing the information and developing control recommendations, the data are fed to the information display subsystem, which by means of the diagrams displays the result of the research. If required, it is possible to create reports on the study of the sample with recommendations using the reporting subsystem.

An analysis of the system on its own can be represented as an analysis of information flows *I_i,j_*. The smallest unit of information flow *I_i,j_* is the image of the metal microstructure f(x,y), which is characterized by a number of parameters concerning the functions $f_{1}\ldots f_{5}$ above.

The set of parameters characterizing the image of the metal microstructure can be considered as an informational description, expressed as follows:

(8) $$I_{i}=\left\{f_{i}\left(x_{i},y_{i}\right),T_{i},E_{i},N_{i},K_{i},r_{i}\right\},i=1,2\ldots n.$$

As it can be seen, from the above parameters, the image of the microstructure of the tested metal sample is characterized by heterogeneous data. At the same time, the set of such data can be used to compile a specific informational description of the image of the metal microstructure. In other words, using the set of these data allows one to create such an informational description of the images of microstructures, which is required by the user at the moment.

**Figure 2:**
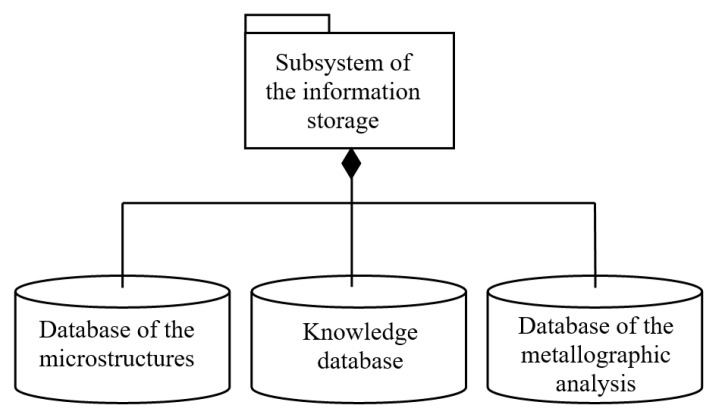
The information storage structure.

From the information description of the microstructure image, it is obviously that the output stream of information is a set of various types of information. Therefore, the information storage subsystem takes the form in Figure 2.

**Figure 3:**
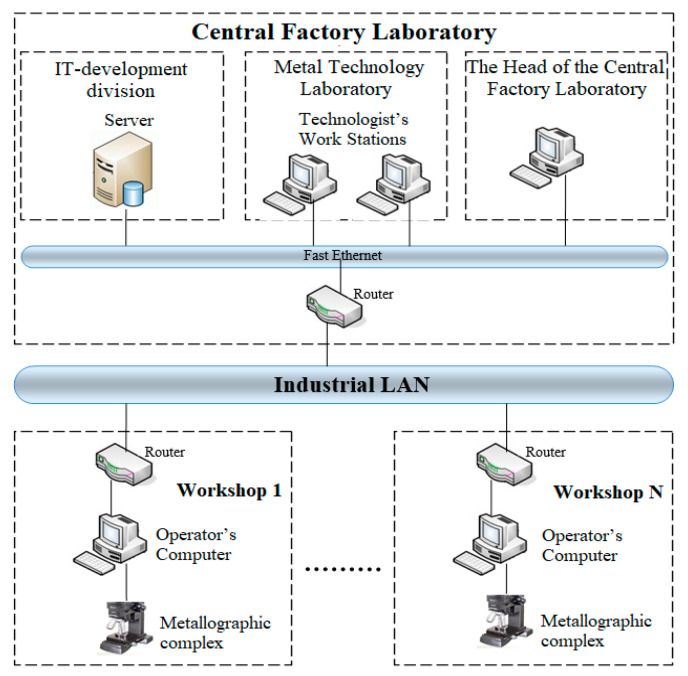
Distributed intelligent metallographic analysis system (deployment model).

The database of microstructures stores images of metal microstructures and their identifiers. The database stores data resulted from analyzing the image of the metal microstructure. The knowledge database is designed to store recommendations regarding the tested metal samples. It is recommended to use the system in a distributed version, as shown in Figure 3.

As shown in Figure 3, depending on the production output, the intelligent system can be implemented according to the client-server architecture or in the local version. If there is no need to use a centralized defect database and integrate the complex into a unified industrial process control system, the local version allows one to implement all the functions of microstructure analysis using the local Database Management System.

According to this model of the system organization, the analysis of metal images can be performed on the server. The results are displayed in the technologist’s computer and recommendations are generated, which in turn are also sent to the server at the central factory laboratory to store and accumulate experience through the local network. Moreover, the nodes of the system can be at a large distance from each other, i.e., the system is scalable. Moreover, in this version of the system organization, there is the addition of new system nodes, for example, for conducting control operations in another workshop of the enterprise, which allows one to talk about the extensibility of the system. 

Due to such organization of the system, it is possible to ensure high efficiency of metallographic analysis in order to reduce resource consumption, as well as the ability to manage the control process remotely, which allows centralized reporting on all control operations carried out at the enterprise.

Thus, the proposed information model reflects the main information flows and their purpose in the process of metallographic analysis, which allows one to go to the stage of developing an intelligent automated system of metallographic quality control of metals, with the capability of data mining.

## 3. Expert Subsystem for the Metallographic Analysis

The major functions of the expert subsystem being developed are as follows:

(1) Acquisition of knowledge, i.e., accumulation of the database of metal microstructures images and their characteristics;
(2) Presentation of knowledge, i.e., presentation of the received information regarding the tested metal sample in a form convenient for the technologist;
(3) Management of the solution search process, i.e., the search for a solution (precedent of metallographic analysis), based on the received information about the metal sample;
(4) Clarification of the decision made, i.e., presentation of the decision or expert conclusion about the tested metal sample in a form convenient for the technologist.

**Figure 4:**
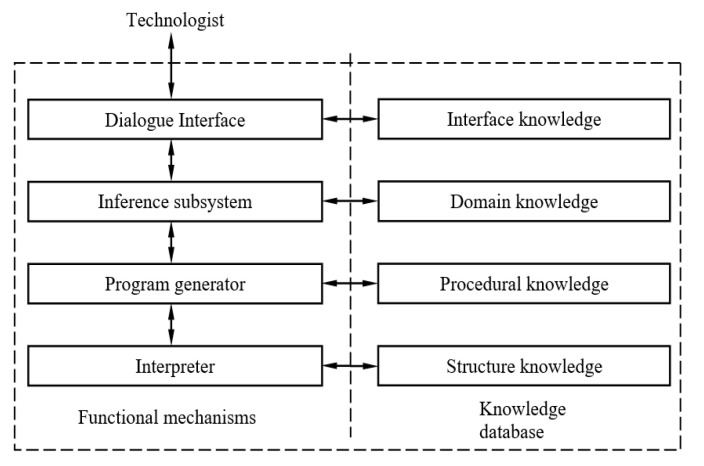
Functional modules of the expert subsystem for metallographic analysis.

The functional model of the expert subsystem that provides the implementation of the functions above is presented in Figure 4.

Within the framework of this functional model, the following functional mechanisms can be distinguished that ensure the operation of the system:

dialogue interface provides communication with the external environment and the conversion of information from external to internal representation and vice versa;

the inference subsystem based on the analysis of the semantics of input information about the metal and the available knowledge about metallographic analysis formulates the statement of the problem, searches for options for solving it and selects the best of them:

- the program generator forms a solver using knowledge of metallographic analysis;
- the interpreter provides the choice and display of an expert reasoning about the tested metal sample.

The knowledge database provides storage and access to various types of knowledge used by an intelligent automated system during its operation.

In the functional model, the following types of knowledge are identified which the system will handle with:

- interface knowledge is the knowledge of interaction with the environment, i.e., about users (technologists) who are allowed access to the system;
- domain knowledge is the knowledge of the domain, representing quantitative and qualitative characteristics of metals, as well as the rules for their evaluation and interpretation;
- procedural knowledge is the knowledge of methods for solving the problem, i.e., information about the type of metallographic analysis and the required expert characteristics of the metal;
- structural knowledge is that about the image of the metal microstructure and expert judgment based on the quantitative characteristics of the metal.

The task of the technologist is to calculate the control parameters required for the operation of the system, the simultaneous change of which during the control process unambiguously evaluates the current situation in the process of metallographic analysis. The group of control parameters includes the following ones:

- grain point (M_G_);
- temper (C);
- defect category (T_d_);
- ferrite/perlite phase ratio (F);
- class of non-metallic inclusions (T_nm_);
- others.

**Figure 5:**
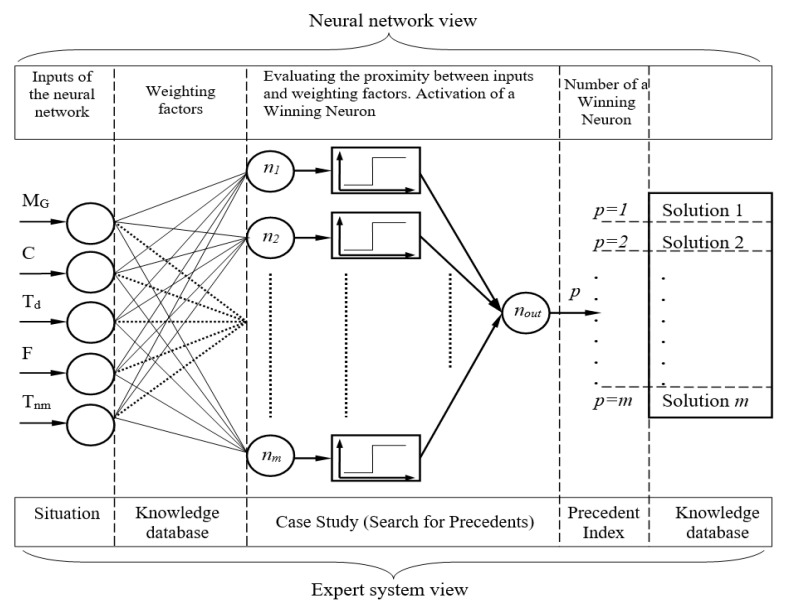
Approach to the presentation of an expert subsystem for metallographic analysis based on a multilayer neural network.

The values of the control parameters are the framework for decision making by the expert subsystem. By analyzing the values of the control parameters, the system takes and generates a solution supplied to the technologist for its implementation. The paper proposes an approach to presenting an expert subsystem based on a multilayer neural network (Figure 5).

As it is obvious from Figure 5, the expert subsystem is based on the multilayer neural network. The number of neurons in the input layer may vary due to the number of control parameters above. The output of the neural network is one neuron, which specifies the precedent index in the knowledge database. To learn the neural network, the back propagation of the error algorithm was chosen [15]. 

As for the set of inputs of the neural network, there are many situations that can arise during the metallographic analysis process. Weighting factors of neurons represent a knowledge database for emerging situations in the process of metallographic analysis. Thus, the task of developing the structure of the knowledge database, as well as the knowledge database management system, is solved, because knowledge is digits that can be stored in any existing database. The process of determining the proximity between the input vector and the weighting factors of the neuron is an evaluation of a close precedent. In other words, by means of a neural network, the precedent closest to the situation has been evaluated. The winning neuron number is the precedent index in the knowledge database. Therefore, this index is the solution index in the knowledge database.

Neural network training was carried out on the basis of reference data of metallographic analysis. The training sample consisted of 160 datasets of control parameters (grain point, temper, defect category, etc.) with 80 of them being “true” group and 80 “damage” group. By “true” the data of reference control parameters are meant, and by “damage” examples data of control parameters distorted by noises are meant, which as a result leads to incorrect classification by a neural network. Thus, the neural network was trained in incorrect classification. To prevent the retraining process [16,17], the training dataset is divided into two sets: training and control ones. As a control sample, 110 datasets of control parameters were used. The data were obtained at the Alchevsk Iron and Steel works.

The authors used two types of neural networks: a multilayer perceptron and a RBF network. A sigmoidal activation function was used for a multilayer perceptron [18].

**Figure 6:**
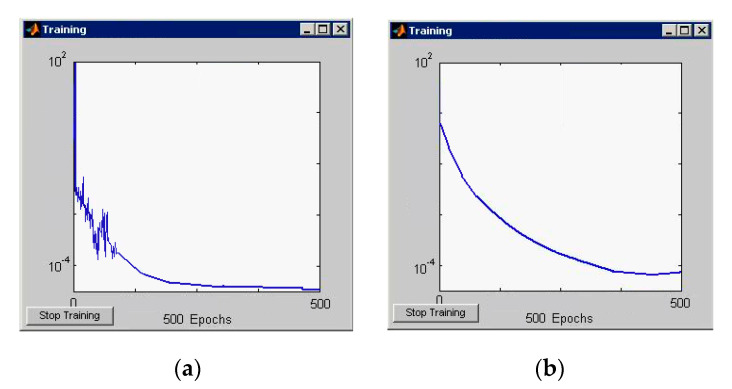
(**a**) Graphs of changes of the learning error (RBF network); (**b**) Graphs of changes of the classification error (RBF network).

**Figure 7:**
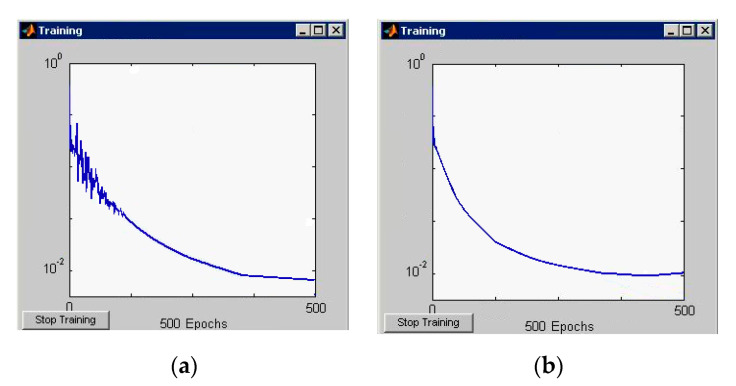
(**a**) Graphs of changes of the learning error (Four-layer perceptron); (**b**) Graphs of changes of the classification error (Four-layer perceptron).

Graphs of changes in learning errors and classification of simulated neural networks are shown in Figure 6 and Figure 7.

The results of experiments for modelling neural networks carried out in MATLAB are shown in Table 1.

As a result, based on the graphs of error changes, the optimal number of training epochs of neural networks was determined using different training algorithms, which amounted to:

- for algorithm “gd”—290 epochs;
- for algorithm “gda”—350 epochs;
- for algorithm “cgb”—270 epochs.

## 4. Development and Research of the Intelligent System for the Metallographic Analysis

**Figure 8:**
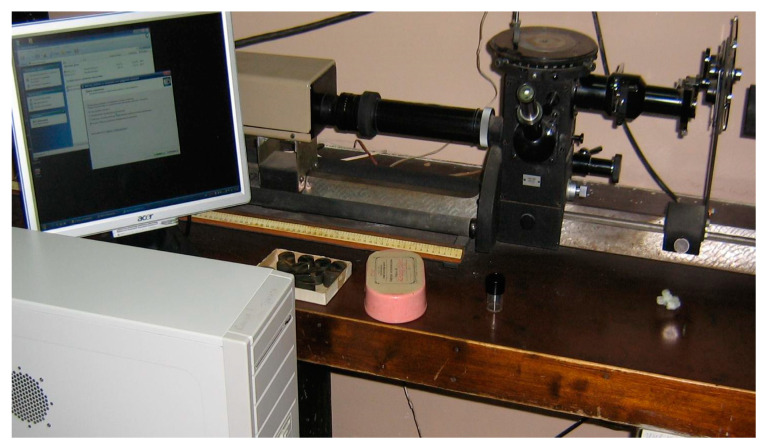
Developed intelligent metallographic analysis system.

Based on the proposed models, an intelligent metallographic analysis system has been developed (Figure 8), which consists of the following components:

- a metallographic complex (microscope and camera with USB-port);
- a personal computer with MetalNeuro software for processing images of metal microstructures and the intellectual analysis of metal data.

**Figure 9:**
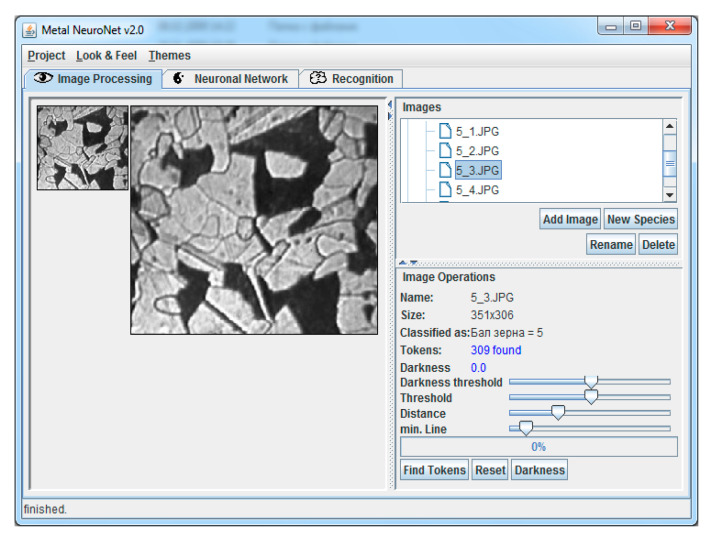
MetalNeuro software for the metallographic analysis (image processing window).

The software part of the system includes as well the software module of the expert subsystem; database; knowledge base. The functions of the MetalNeuro software in the analysis mode are the automatic processing of a metal image, the function responsible for metal data analysis and the function responsible for the database creation and a knowledge database by registering all the parameters of every image of the metal microstructure. The “Image Processing” and “Recognition” tabs of specialized software (Figure 9) are intended for the automatic processing of a metal image. 

The “Project” menu is intended for realizing the expert subsystem functions. The “Neural Network” tab is intended for realizing the neural network subsystem. The software supports creation of a neural network and setting its main parameters (number of layers, neurons, learning rule, etc.) and loading of data on the metallographic analysis for training the neural network.

**Figure 10:**
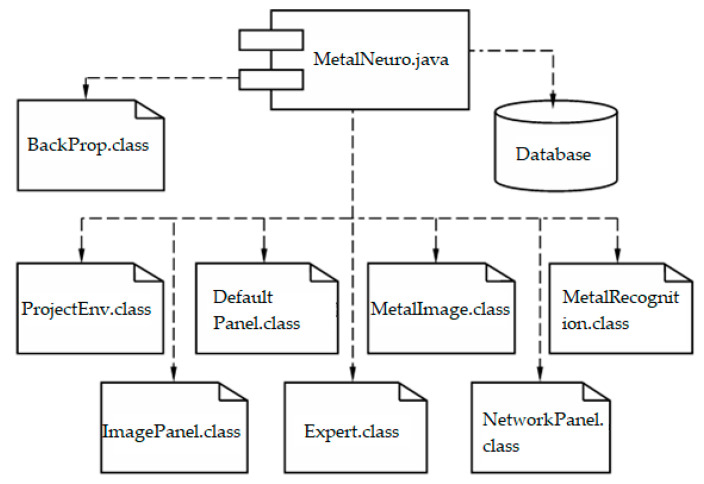
The physical structure of the MetalNeuro software for metallographic analysis.

To develop the MetalNeuro software the Java language and Eclipse IDE for Enterprise Java Developers were used. The component model (physical structure) of the MetalNeuro software for metallographic analysis is presented in Figure 10.

The “MetalNeuro.java” component implements a certain set of interfaces for interconnection with the database and processing modules to automate metallographic analysis.

The “ProjectEnv.class” contains information about the up-to-date state of the system.

The “DefaultPanel.class” describes the main panel of the GUI (Graphic User Interface).

The “MetalImage.class” implements properties and methods for accessing the current image being processed.

The “MetalRecognition.class” connects all modules, ensuring the functioning of information flows.

The “ImagePanel.class” contains class methods for visualizing recognition results.

The “NetworkPanel.class” contains a class that inherits class methods for visual operation within a neural network.

The “Expert.class” contains the methods, properties and functions required for the expert system.

The “BackProp.class” implements multilayer neural network, as well as the error back propagation algorithm (Algorithm 1).
**Algorithm 1.** A part of the “BackProp.class” code1:  public class BackProp2:  {3:  private double inputA[];     // activations input4:  private double hiddenA[];  // activations hidden5:  private double hiddenN[];  // sum of products for hidden units6:  private double hiddenD[];  // output error7:  private double hiddenW[][];  // connection weights matrix8:  private double outputA[];  // activations output9:  private double outputN[];  // sum of products10:private double outputD[];  // output error11:private double outputW[][]; // connection weights matrix12:private int   numInput;// number of neurons on input layer13:private int   numHidden;// number of neurons on hidden layer14:private int   numOutput;// number of neurons on output layer15:private int epoch; // number of epochs of the learn process16:private double momentum; // momentum17:private double alpha; // learnrate18:private double absError=0.0; // the absolute error of the learning proc.19:private double sigmoidDeriv(double x) /** sigmoid activation function */20:{21:return (sigmoid(x) * (1 − sigmoid(x)));22:}23:private void feedForward() /* method to do the feed forward */24:{25:// calculate the hidden weights26:for(int i = 0; i < numHidden; i++)27:{28:sum2 = biasH[i];29:for(int j=0; j < numInput; j++)30:sum2 += hiddenW[i][j]* inputA[j];31:hiddenN[i] = sum2;32:hiddenA[i] = sigmoid(sum2);33:}34:// calculate the new output weights35:for(int i = 0; i < numOutput; i++)36:{37:sum2 = biasO[i];38:for(int j = 0; j < numHidden; j++)39:sum2 += outputW[i][j]* hiddenA[j];40:outputN[i] = sum2;41: }42:}43:private void updateWeights()  /** method to update the weights */44:{45:double sum2;46:for(int j = 0; j < numHidden; j++)47:{48:sum2 = 0.0;49:for(int i = 0;i < numOutput; i++)50:sum2 += outputD[i]* outputW[i][j];51:sum2 *= sigmoidDeriv(hiddenN[j]);52:biasH[j] += sum2 * alpha;53:for(int i=0; i < numInput; i++)54:hiddenW[j][i] += alpha * sum2 * inputA[i];55:}56:}57:}

The world leaders in the production of PLCs are Siemens (https://new.siemens.com/, Germany), Rockwell Automation (https://www.rockwellautomation.com/en-us.html, USA), Schneider Electric (https://www.se.com/, USA) and GE Intelligent Platforms (https://www.ge.com/, USA). Russian Aries (Russia) and Segnetics (Saint-Petersburg Saint-Petersburg, Russia) controllers are not much inferior to their foreign counterparts, but they differ in a more favorable price. There are five main programming languages for programming the controllers according to the IEC 61131 standard. Three of the five languages are graphical—LD, FBD, SFC, and two are text—ST and IL [19,20,21,22]. The results of the functioning of the developed system for assessing the quantitative characteristics of the steel 10ChSND(S420N) are summarized in Table 2.

## 5. Discussion

Analysis of the results in Table 2 indicates a high probability of correct steel image recognition (more than 92%) and as a result a high quality of assessing the quantitative characteristics of steels using the neural network approach and developed software. The probability of correct steel image recognition was 83% before developing new automated system and applying the neural network approach. The evaluating of the quantitative characteristics of steel 10ChSND(S420N) by developed system was at Alchevsk Iron and Steel Works (Alchevsk, Ukraine).

In the future, the neural network approach and developed system can be effectively used to assess a wide class of objects in the metallurgical industry, for example, to assess the state of the pipes, long steel products etc. 

## 6. Conclusions

Thus, the following results were obtained:

(1) An information model is proposed, which represents an intelligent system of metallographic analysis in the form of a set of subsystems, the interaction of which ensures the performance of metallographic analysis functions.
(2) The deployment model of an intelligent metallographic analysis system is proposed and described.
(3) The expert subsystem, implemented on the basis of the proposed neural network, allows one to bring the process of metallographic analysis to a whole new level.
(4) An intelligent metallographic analysis system with MetalNeuro software are developed.

## Figures and Tables

**Table 1:** The results of the experiments carried out in MATLAB.

Type of A Neural Network	Number of Training Epochs	Training Algorithm	Classification	
			Ok	Error
RBF network	500	gd	93.5	3.5
RBF network	500	gda	95.6	1.8
RBF network	500	cgb	93.4	3.9
Four-layer perceptron	500	gd	91.6	5.4
Four-layer perceptron	500	gda	91.5	4.9
Four-layer perceptron	500	cgb	92.8	4.5

**Table 2:** The results of assessing the quantitative characteristics of steel 10ChSND(S420N).

Characteristics of Steel 10ChSND (S420N)	The Total Amount of the Steel Images	The Number of Correct Recognized Steel Images	The Full Probability of Correct Alloy Image Recognition, %
Grain point	231	224	93.1
Martensite/troostite phase ratio	121	118	95.6
Ferrite/Perlite Phase Ratio	121	119	92.3
Sulphide point	142	133	94.2
Silicate point	142	134	93.6
Point of stitched (line) nitrides	142	134	93.9
